# Response of Methanogenic Microbial Communities to Desiccation Stress in Flooded and Rain-Fed Paddy Soil from Thailand

**DOI:** 10.3389/fmicb.2017.00785

**Published:** 2017-05-05

**Authors:** Andreas Reim, Marcela Hernández, Melanie Klose, Amnat Chidthaisong, Monthira Yuttitham, Ralf Conrad

**Affiliations:** ^1^Max Planck Institute for Terrestrial MicrobiologyMarburg, Germany; ^2^Centre for Biological Sciences, University of SouthamptonSouthampton, UK; ^3^Joint Graduate School of Energy and Environment, King Mongkut’s University of Technology ThonburiBangkok, Thailand; ^4^Faculty of Environment and Resource Studies, Mahidol UniversitySalaya, Thailand

**Keywords:** methanogenesis, rice field, flooding management, desiccation stress, archaea, bacteria, mcrA

## Abstract

Rice paddies in central Thailand are flooded either by irrigation (irrigated rice) or by rain (rain-fed rice). The paddy soils and their microbial communities thus experience permanent or arbitrary submergence, respectively. Since methane production depends on anaerobic conditions, we hypothesized that structure and function of the methanogenic microbial communities are different in irrigated and rain-fed paddies and react differently upon desiccation stress. We determined rates and relative proportions of hydrogenotrophic and aceticlastic methanogenesis before and after short-term drying of soil samples from replicate fields. The methanogenic pathway was determined by analyzing concentrations and δ^13^C of organic carbon and of CH_4_ and CO_2_ produced in the presence and absence of methyl fluoride, an inhibitor of aceticlastic methanogenesis. We also determined the abundance (qPCR) of genes and transcripts of bacterial 16S rRNA, archaeal 16S rRNA and methanogenic *mcrA* (coding for a subunit of the methyl coenzyme M reductase) and the composition of these microbial communities by T-RFLP fingerprinting and/or Illumina deep sequencing. The abundances of genes and transcripts were similar in irrigated and rain-fed paddy soil. They also did not change much upon desiccation and rewetting, except the transcripts of *mcrA*, which increased by more than two orders of magnitude. In parallel, rates of CH_4_ production also increased, in rain-fed soil more than in irrigated soil. The contribution of hydrogenotrophic methanogenesis increased in rain-fed soil and became similar to that in irrigated soil. However, the relative microbial community composition on higher taxonomic levels was similar between irrigated and rain-fed soil. On the other hand, desiccation and subsequent anaerobic reincubation resulted in systematic changes in the composition of microbial communities for both Archaea and Bacteria. It is noteworthy that differences in the community composition were mostly detected on the level of operational taxonomic units (OTUs; 97% sequence similarity). The treatments resulted in change of the relative abundance of several archaeal OTUs. Some OTUs of *Methanobacterium, Methanosaeta*, *Methanosarcina*, *Methanocella* and *Methanomassiliicoccus* increased, while some of *Methanolinea* and *Methanosaeta* decreased. Bacterial OTUs within *Firmicutes*, *Cyanobacteria, Planctomycetes* and *Deltaproteobacteria* increased, while OTUs within other proteobacterial classes decreased.

## Introduction

Microbial degradation of organic matter to CH_4_ and CO_2_ usually occurs in soils that are anoxic and depleted in electron acceptors such as nitrate, ferric iron and sulfate. In paddy soils such conditions are met some time after flooding, the time period depending on the relative availability of organic electron donors and inorganic electron acceptors (Yao et al., 1999). Then, CH_4_ is produced by a complex microbial community consisting of hydrolytic, fermenting and methanogenic microorganisms. These microbes degrade organic matter to acetate, and H_2_ and CO_2_, which are then converted to CH_4_ by aceticlastic and hydrogenotrophic methanogenesis, respectively (Conrad, 2007). Although this microbial community typically operates under anaerobic conditions, i.e., being active when the environment is anoxic and reduced, members of the community can be more or less tolerant to oxic and aerobic conditions. Thus, methanogenic archaeal species were found to survive in aerated soils and also survive desiccation (Fetzer et al., 1993). This is also true for hydrolytic and fermenting bacteria. *Firmicutes*, for example, form endospores to survive detrimental conditions.

It is probably the microbial plasticity that guarantees methanogenic function in a large variety of different soil environments. Thus, methanogenic activity was found in virtually all soils tested, including desert soils (Peters and Conrad, 1996; Angel et al., 2012a). However, the structures (i.e., abundance and composition) of the various methanogenic communities were sometimes quite different. Thus, community structures were found to be different when comparing soils that are permanently flooded (lake sediments) (Mandic-Mulec et al., 2012; Youngblut et al., 2014; Ji et al., 2016), seasonally flooded (irrigated rice paddies) (Lueders and Friedrich, 2000; Peng et al., 2008; Rui et al., 2009; Kim and Liesack, 2015), occasionally flooded (rice rotated with upland crops) (Fernandez Scavino et al., 2013; Lopes et al., 2014; Liu et al., 2015; Breidenbach et al., 2016a), or rarely flooded (upland soils) (Angel et al., 2012a,b; Hernández et al., 2017). However, rice paddies that are flooded by irrigation or by rain have not yet been compared.

The methanogenic function of soil microbial communities has been shown to exhibit a remarkable resilience to short term desiccation. Such desiccation causes stress to the soil microbial community because of decrease of water activity and because of aeration, which provides potentially toxic O_2_ (Fetzer et al., 1993; Erkel et al., 2005) and allows the regeneration of inorganic electron acceptors (e.g., nitrate, ferric iron, sulfate) (Klüber and Conrad, 1998; Ratering and Conrad, 1998). However, very little is known about the microbial background of such resilience, in particular to which extent the microbial community structures are affected (Conrad et al., 2014b; Ji et al., 2015; Hernández et al., 2017).

Therefore, we studied soil from rice paddies in central Thailand, which were on the same soil series, but were managed as either irrigated or rain-fed fields. We were interested whether function and structure of archaeal and bacterial methanogenic communities were different in these soils and how their communities reacted upon short-term desiccation.

## Materials and Methods

### Sampling

Paddy soil was collected in December 2012 from irrigated and rain-fed rice fields in Ang Thong province, Central Thailand (Supplementary Figure S1). All fields were on the identical soil series, i.e., Nakhon Pathom series (Np), which is an Aeric Tropaqualf with a clay texture (Pisoot and Hari, 2001). The mean precipitation in Ang Thong province is 4–45 mm during the dry season (November to April) and 90–240 mm during the wet season (May to October). The irrigation system was introduced around 1950. Both irrigated and rain-fed rice is cultivated in bunded fields. Irrigated rice has two cropping seasons (dry and wet), during which the fields are artificially flooded. Rain-fed rice fields, which are flooded only by rain during the wet season, have one cropping season (dry season). Rain-fed rice fields are characterized by lack of water control. At the time of sampling, the rice fields were in the early stage of seasonal development, some being bare, others planted and some with rice already grown to the tillering stage. In 2011, 1 year before sampling, Thailand experienced a flood catastrophe, starting with the monsoon period end of July 2011 and lasting until January 2012. During this time, rain-fed rice fields were also completely submerged. Irrespectively, fields for sampling were selected on the basis whether they had been managed as irrigated or rain-fed fields in the past. Four different fields (size about 1 ha) of each management type were sampled. Soil samples were taken in triplicate and then mixed to give a composite sample for each replicate field. Soils were transported in fresh state to the Max Planck Institute in Marburg, then immediately frozen and stored at -18°C until setup of the experiments about 3 months later. Chemical analyses (**Table 1**) were done as described before (Conrad and Klose, 2006).

**Table 1:** Main characteristics of paddy soils sampled from irrigated and rain-fed rice fields (mean ± SE, *n* = 4).

	Irrigated	Rain-fed
Location	14.68°N 100.28°E	14.64°N 100.23°E
Org. C (%)	1.86 ± 0.13	2.34 ± 0.05
Total N (%)	0.18 ± 0.01	0.21 ± 0.01
C/N	10.4	11.2
δ^13^C_org_ (‰)	-25.08 ± 0.39	-27.15 ± 0.22
pH	6.9 ± 0.1	6.7 ± 0.1


### Incubation Conditions

The incubation procedure was as described by Ji et al. (2015), briefly outlined in Supplementary Figure S2. Fresh soils were used for anoxic soil slurries. To mimic flooding periods, 10 g fresh soil was placed into a 26-ml glass pressure tube. The dry weight was determined at the end of the experiment by drying and weighing. The average soil water contents were 79 and 83% (per 100 g dry soil) for irrigated and rain-fed soils, respectively. Slurries were incubated at 25°C for 1 month, afterward slurries were dried under air for 2 weeks at 35°C (drainage period). To mimic reflooding, the dried soil (6 g) was mixed with 6 ml sterile anoxic H_2_O and incubated in 26-ml glass pressure tubes at 25°C for another 7 weeks. Tubes were closed with a butyl rubber stopper and incubated (without shaking) under a N_2_ atmosphere until CH_4_ production was constant. Methyl fluoride, an inhibitor for aceticlastic methanogenesis, was added (3%) as a control treatment (Janssen and Frenzel, 1997). All incubations were performed using samples collected from four replicate fields each of irrigated and rain-fed fields.

### Gas Measurements

The chemical analysis of gas and liquid samples was done as described before (Conrad et al., 2014b). Briefly, CH_4_ and CO_2_ were analyzed by gas chromatography (GC) and the δ^13^C by either GC combustion isotope ratio mass spectrometry (GC-C-IRMS). The δ^13^C of organic matter was analyzed by the Centre for Stable Isotope Research and Analysis (KOSI) at the University of Göttingen using an elemental analyzer coupled to an IRMS. The fraction (*f_H2_*) of CH_4_ production by hydrogenotrophic methanogenesis was calculated by mass balance as described before (Conrad et al., 2010) using

$$f_{H2}=\frac{\left(\delta^{13}C_{CH4}-\delta^{13}C_{CH4}-ma\right)}{\left(\delta^{13}C_{CH4}-mc-\delta^{13}C_{CH4}-ma\right)}$$

with δ^13^C_CH4_ = δ^13^C of total CH_4_ produced, δ^13^C_CH4-mc_ = δ^13^C of CH_4_ produced from hydrogenotrophic methanogenesis, which is equivalent to the CH_4_ produced in the presence of CH_3_F, and δ^13^C_CH4-ma_ = δ^13^C of CH_4_ produced from aceticlastic methanogenesis. The δ^13^C_CH4-ma_ was approximated from the δ^13^C of soil organic matter assuming that δ^13^C_CH4-ma_ = δ^13^C_org_-10 (Conrad et al., 2014a).

### DNA and RNA Extraction and qPCR

Total nucleic acid was extracted using a modified version of the protocol of Bürgmann et al. (2001) as described elsewhere (Breidenbach and Conrad, 2015). In short, cells were disrupted by bead beating and nucleic acid was purified using phenol/chloroform extraction. For RNA analysis a subset of total nucleic acid was treated with DNase, checked for complete DNA digestion by bacterial 16S rRNA PCR and reverse transcribed to cDNA using random hexamers.

The abundance of bacterial 16S rRNA, archaeal 16S rRNA and of methanogenic *mcrA* genes was determined by qPCR with primer sets Ba519f/Ba907r (Stubner, 2002), Arch364-f/934b-r and mlas-mod-f/mcrA-rev-r, respectively (Kemnitz et al., 2005; Angel et al., 2012a). The qPCR was conducted in 96-well plates using a CFX cycler (BioRad). PCR reactions of bacterial 16S rRNA, archaeal 16S rRNA contained in a total volume of 25 μl 1 × SYBRGreenReadyMix (Sigma), 0.33 μM of each primer, 4 mM MgCl_2_ (Sigma) and 3 mM MgCl_2_ (Sigma), respectively. For *mcrA* the reactions contained in 15 μl total volume 1× SsoFast EvaGreen supermix (BioRad) and 0.4 μM primer each. All qPCR reactions contained 1–2 μl target DNA or cDNA, respectively. The conditions were as follows: for bacterial 16S rRNA gene: 8 min at 94°C, 40 cycles of 94°C for 20 s, 50°C for 20 s, 72°C for 45 s, 75°C for 15 s; for archaeal 16S rRNA gene: 6 min at 94°C, 40 cycles of 94°C for 35 s, 66°C for 30 s, 72°C for 45 s, 86.5°C for 10 s; and for *mcrA* gene: 5 min 94°C, 40 cycle at 95°C for 30 s, 57°C for 45 s, 72°C for 30 s, 84°C for 10 s. For bacterial 16S rRNA genes efficiencies of 68.0–74.6%, for archaeal 16S rRNA genes efficiencies of 86.2–97.2% and for *mcrA* genes efficiencies of 88.2–93.3% with *R*^2^ values > 0.99 were obtained. Technical duplicates were performed for each of the replicates. To test the significance of the differences between irrigated and rain-fed soils, two-tailed independent *t*-tests were applied using Microsoft Excel version 15.17. Terminal restriction fragment length polymorphism (T-RFLP) and cloning and sequencing for archaeal 16S rRNA gene and *mcrA* gene are detailed in Appendix S1 (Supplementary Information).

### Illumina Library Preparation and Sequencing

PCR primers (515F, 5′-GTGCCAGCMGCCGCGGTAA-3′ and 806R, 5′-GGACTACVSGGGTATCTAAT-3′) targeting the V4 region of the 16S rRNA gene (approximately 250 nucleotides) for both Archaea and Bacteria were used (Bates et al., 2011). Individual PCRs contained a 6-bp molecular barcode integrated in the forward primer. PCR conditions consisted of an initial denaturation at 94°C for 5 min, followed by 28 cycles of 94°C for 30 s, 50°C for 30 s, and 68°C for 30 s and a final extension at 68°C for 10 min (Hernández et al., 2015). Amplicons were purified using a PCR cleanup kit (Sigma) and quantified using a Qubit 2.0 fluorometer (Invitrogen). Finally, samples were pooled in an equimolar concentration and sequenced on separate runs for MiSeq using a 2 bp × 300 bp paired end protocol. Library preparation and sequencing was performed at the Max Planck Genome Centre (MPGC), Cologne, Germany. Supplementary Table S1 in the supplemental material summarizes the barcode sequences for each of the samples.

### Bioinformatic Analyses

The first step was quality filtering and trimming forward and reverse adaptors from the sequences using the tool cutadapt (Martin, 2011). Secondly, forward and reverse reads were merged by using the usearch fastq_mergepairs command (Edgar, 2013). Downstream processing was performed by using UPARSE (Edgar, 2013) and UCHIME pipelines (Edgar et al., 2011). Briefly, sequences shorter than 250 bp were discarded, singletons were retained, and operational taxonomic units (OTUs) were defined at a sequence identity level of 97%.

### Statistical Analyses and OTU Classification

Statistical analyses were performed using the vegan package (Oksanen et al., 2013) in R software version 3.0.1.^1^ For all OTU-based statistical analyses, the data set was normalized by a Hellinger transformation (Legendre and Gallagher, 2001) using the *decostand* function. For beta-diversity, non-metric multidimensional scaling (NMDS) ordination of Hellinger distances was carried out using the *cmdscale* function. Heatmaps were constructed with the gplots (Warnes et al., 2015) for the OTUs explaining most of the differences between samples. Principal component analysis (PCA) of the Hellinger transformed data was performed using the *prcomp* function. The 50 OTUs explaining most of the differences between samples were defined as the OTUs contributing the largest absolute loadings in the first and second dimensions of the PCA (Breidenbach et al., 2016a; Hernández et al., 2017), obtained from the rotation output file. Hierarchical clustering of the distance matrix was carried out with the “ward” method using *hclust* function. The heatmap was constructed using the *heatmap.2* function in gplots package (Hernández et al., 2017). For the T-RFLP analysis TRF sizes were rounded to integers and treated as OTUs. Overall signal intensity was standardized according to Dunbar et al. (2001) and expressed as fractions. The NMDS (using the function metaMDS and Bray–Curtis dissimilarity index) were done with the vegan package, version 2.3-0 (Oksanen et al., 2015).

### Taxonomy Analysis

A representative sequence of each OTU was aligned against the SILVA 16S rRNA gene database using the naïve Bayesian classifier (bootstrap confidence threshold of 80%) by using the mothur software platform (Schloss et al., 2009).

### Data Accession

Sequence data were deposited in the NCBI Sequence Read Archive (SRA) in two separate Bio-projects. Irrigated rice field soil samples under accession number PRJNA362529 and rain-fed rice fields soil samples under accession number PRJNA362531.

## Results

### Functional Characteristics and Microbial Abundance

**FIGURE 1:**
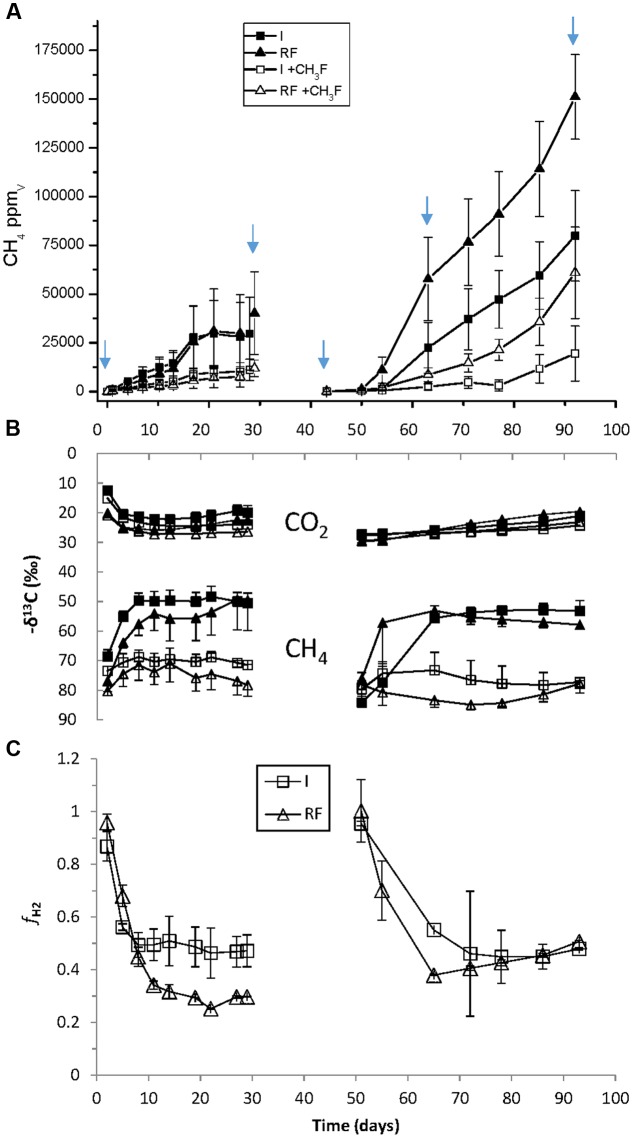
**Methanogenesis in anaerobic incubations of paddy soil from irrigated (I) and rain-fed (RF) rice fields in the absence and presence of 3% methyl fluoride.** **(A)** Production of CH_4_ (1 ppmv ≈ 16/24.47/6 = 0.11 nmol gdw^-1^); **(B)** δ^13^C of produced CH_4_ and CO_2_; **(C)** fraction (*f*_H2_) of CH_4_ produced from CO_2_. The arrows indicate when samples for molecular analysis were taken. The sample at day 63 was only used for qPCR and T-RFLP, not for Illumina sequencing. Mean ± SE, *n* = 4 different fields.

The main chemical characteristics of the paddy soils from irrigated and rain-fed rice fields were similar (**Table 1**). In addition, both soils initially exhibited the same rates of CH_4_ production (5.3 nmol h^-1^ gdw^-1^) (**Figure 1A**). However, the δ^13^C of CH_4_ and CO_2_ in the presence and absence of methyl fluoride, an inhibitor of aceticlastic methanogenesis, were slightly different (**Figure 1B**). These data resulted in a percentage of CH_4_ produced from CO_2_ that was lower in rain-fed (ca. 30%) than in irrigated soil (ca. 45%) (**Figure 1C**). After desiccation and subsequent reincubation under anaerobic conditions, CH_4_ production was stimulated in both soils, but was higher in rain-fed (15.2 nmol h^-1^ gdw^-1^) than in irrigated (8.3 nmol h^-1^ gdw^-1^) soil (**Figure 1A**). However, the contribution of hydrogenotrophic methanogenesis to CH_4_ production was in both soils about 45% (**Figure 1C**).

**FIGURE 2:**
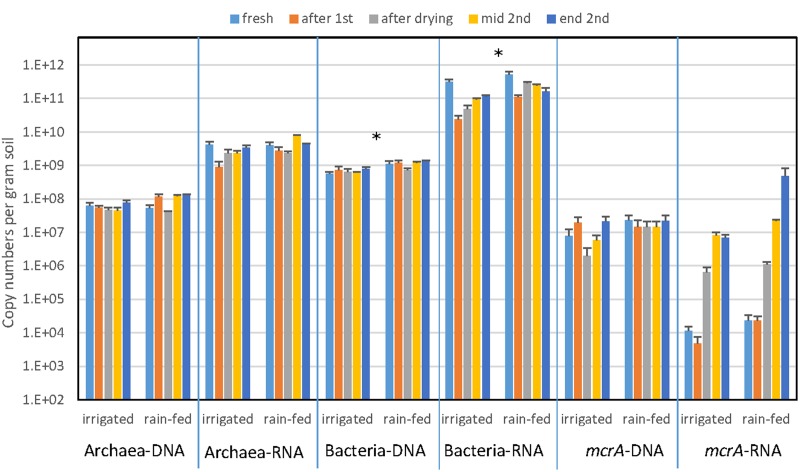
**Copy numbers of genes (DNA) and transcripts (RNA) of archaeal and bacterial ribosomal RNA and methanogenic *mcrA* in paddy soil from irrigated and rain-fed rice fields.** The copy numbers were measured in fresh soil, after the first anaerobic incubation, after desiccation, and after the subsequent second anaerobic reincubation (mid term and in the end) as indicated by arrows in **Figure 1**. Mean ± SE, *n* = 4 different fields. The differences between irrigated and rain-fed soils were tested by two-tailed independent *t*-tests, indicated by ^∗^ when *P* < 0.05.

The abundance of archaea and bacteria was determined by PCR targeting the gene copies of archaeal and bacterial 16S rRNA. In addition, we also determined the abundance of 16S rRNA copies as proxy for ribosome abundance in the archaeal and bacterial communities. The gene copy numbers of Bacteria were about a magnitude larger than those of Archaea, but both were similar for irrigated and rain-fed soil and were also similar for the different treatments (**Figure 2**). Significant differences comparing irrigated and rain-fed soils were found only in Bacteria, both the 16S rRNA gene (*P* = 0.008) and the ribosomal RNA (*P* = 0.01). Copy numbers of 16S rRNA were 1–2 orders of magnitude higher than copy numbers of 16S rRNA gene both for Archaea and Bacteria (**Figure 2**). The abundance of methanogens was determined by measuring the gene copy numbers of *mcrA*, which were about one order of magnitude lower than those of the archaeal 16S rRNA genes. Gene copy numbers of *mcrA* were again similar for irrigated and rain-fed soils and were similar between the different treatments. However, transcript copy numbers of *mcrA* increased dramatically (2–4 orders of magnitude) after desiccation and reincubation (**Figure 2**).

### Composition of the Archaeal Community

**FIGURE 3:**
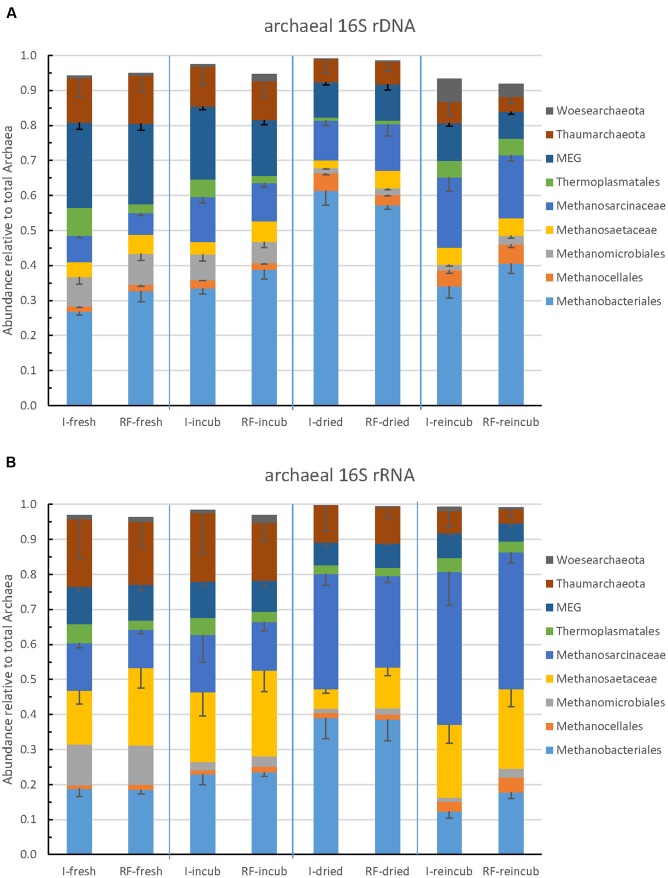
**Relative abundance of the predominant archaeal taxa (phyla, orders, or families) in paddy soil from irrigated (I) and rain-fed rice (RF) fields determined in fresh soil (-fresh), after anaerobic incubation (-incub), after desiccation (-dried), and in the end of the subsequent anaerobic reincubation (-reincub).** **(A)** 16S rRNA genes and **(B)** 16S rRNA. Mean ± SE, *n* = 4 different fields.

The community composition of Archaea was determined by Illumina sequencing and T-RFLP analysis of archaeal 16S rRNA genes. The relative abundance of the major archaeal taxa representing together >90% of the total number of archaeal sequences obtained by Illumina sequencing are shown in **Figure 3**. *Methanobacteriales* represented about 25–60% of the Archaea, followed by *Methanosarcinaceae* (5–20%), *Methanosaetaceae* and *Methanocellales* and *Methanomicrobiales*. The rest of the Archaea (20–50%) belonged to the euryarchaeota *Thermoplasmatales*, the Miscellaneous Euryarchaeota Group (MEG), and the phyla *Thaumarchaeota* and *Woesearchaeota* (**Figure 3A**). The relative abundances of these taxa were strikingly similar between irrigated and rain-fed soils, but changed between the different treatments. Thus, the relative abundance of *Methanobacteriales* was highest after desiccation and that of *Methanosarcinaceae* was highest after desiccation and rewetting (**Figure 3A**). These tendencies were even more pronounced when comparing the relative ribosome abundances (**Figure 3B**). Again, irrigated and rain-fed soils showed similar composition of the higher (families to phyla) archaeal taxa, while soil treatment showed in particular an increase of the relative abundance of *Methanosarcinaceae* after desiccation and rewetting.

**FIGURE 4:**
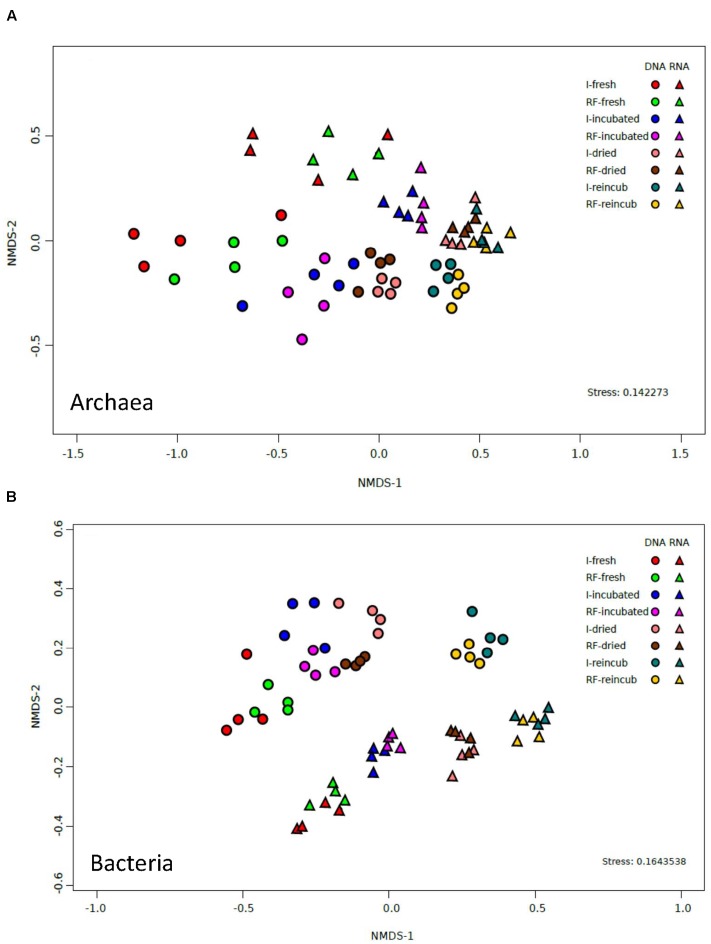
**NMDS plots of** **(A)** archaeal OTUs and **(B)** bacterial OTUs derived from both 16S rDNA and 16S rRNA extracted from soil of four replicate irrigated (I) and rain-fed (RF) rice fields; in fresh soil, after anaerobic incubation, after desiccation (dried), and after anaerobic reincubation (reincub).

The similarity between irrigated and rain-fed soils and the dissimilarities between the different treatments were confirmed by analyzing the relative abundance of OTUs of archaea. NMDS of the archaeal OTUs derived from either 16S rRNA genes or from the ribosomal RNA showed no clear separation between the OTUs from irrigated and rain-fed soils (**Figure 4A**). However, there was a separation according to whether the OTUs were derived from DNA or from RNA. Furthermore, there was a tendency to separate OTUs according to whether the samples were from fresh, incubated, desiccated and reincubated soils (**Figure 4A**).

**FIGURE 5:**
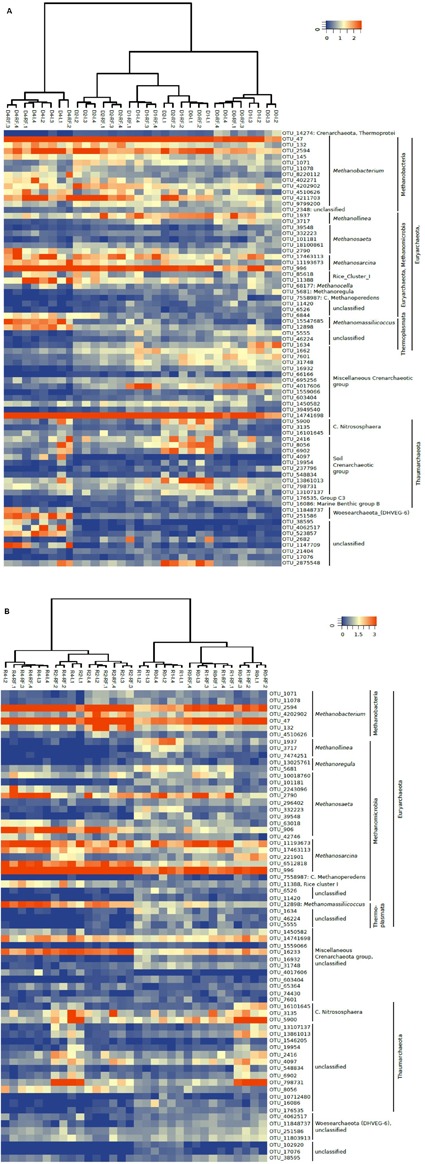
**Heatmap of the most relevant archaeal OTUs derived from** **(A)** 16S rDNA (labeled D) and **(B)** 16S rRNA (labeled R) extracted from soil of four replicate irrigated (labeled I.1–I.4) and rain-fed (labeled RF.1–RF.4) rice fields; in fresh soil (D0 or R0), after anaerobic incubation (D1 or R1), after desiccation (D2 or R2), and after anaerobic reincubation (D4 or R4). The colored scale gives the percentage abundance of OTUs relative to total Archaea.

A heatmap of the most relevant OTUs also showed that these clustered relatively well along the soil treatments, but showed no significant difference between irrigated and rain-fed soils. This was the case both for 16S rDNA (**Figure 5A**) and 16S rRNA (**Figure 5B**). However, the heatmaps did show a clear clustering according to treatment. On the DNA level, OTUs after the desiccation and reincubation were clearly separate from the other treatments (**Figure 5A**). On the RNA level, OTUs before and after desiccation formed clearly separate clusters (**Figure 5B**). A closer look shows that several archaeal OTUs increased in relative abundance (especially of ribosomal RNA) after desiccation, e.g., the OTU_132 and OTU_4202902 within the *Methanobacteria*, OTU_906 (not on the gene level) within the *Methanosaeta*, OTU_17463113 and OTU_6512818 (not on the gene level) within the *Methanosarcina*, OTU_11388 within *Methanocella* (Rice cluster I), OTU_12898 (on the gene level also OTU_15547685) within *Methanomassiliicoccus*, and OTU_14741698 and OTU_16233 (not on the gene level) within the miscellaneous Crenarchaeota group (MCG). On the other hand, several OTUs decreased after desiccation, e.g., all those belonging to *Methanolinea*, and several belonging to *Methanosaeta* (OTU_332223 and OTU_39548).

The archaeal community composition was also assessed by T-RFLP fingerprinting of 16S rDNA (Supplementary Figure S3) and rRNA (Supplementary Figure S4). These fingerprints also showed the presence of *Methanobacteriales*, *Methanosarcinaceae*, *Methanosaetaceae* and *Methanocellales*. Consistently, NMDS plots of the T-RFLP data did not differentiate well between the different soils (Supplementary Figure S5). However, they also did not differentiate well between the different treatments (Supplementary Figure S5).

The methanogenic community was assessed by T-RFLP fingerprinting of *mcrA* genes (Supplementary Figure S6) and transcripts (Supplementary Figure S7). Both showed the presence of putative *Methanobacteriales*, *Methanosarcinaceae*, and *Methanosaetaceae*. NMDS could differentiate the *mcrA* gene communities before and after desiccation, and to some extent also between irrigated and rain-fed soil (Supplementary Figure S8). However, the *mcrA* transcripts were only well differentiated between the fresh soil and the incubated soils, but not between irrigated and rain-fed soil and between the soil treatments (Supplementary Figure S8). Also the patterns of *mcrA* transcripts were quite different from those of *mcrA* genes, with strong increase of the relative abundance of T-RF of 147 bp (presumably *Methanosaetaceae*) and the disappearance of T-RF of 468 bp (presumably *Methanosarcinaceae*) in the transcripts (Supplementary Figures S6, S7). However, *Methanosarcinaceae* (represented by T-RFS of 391 and 423 bp) as well *Methanobacteriaceae* (T-RF of 504 bp) were found in both *mcrA* genes and transcripts.

### Composition of the Bacterial Community

**FIGURE 6:**
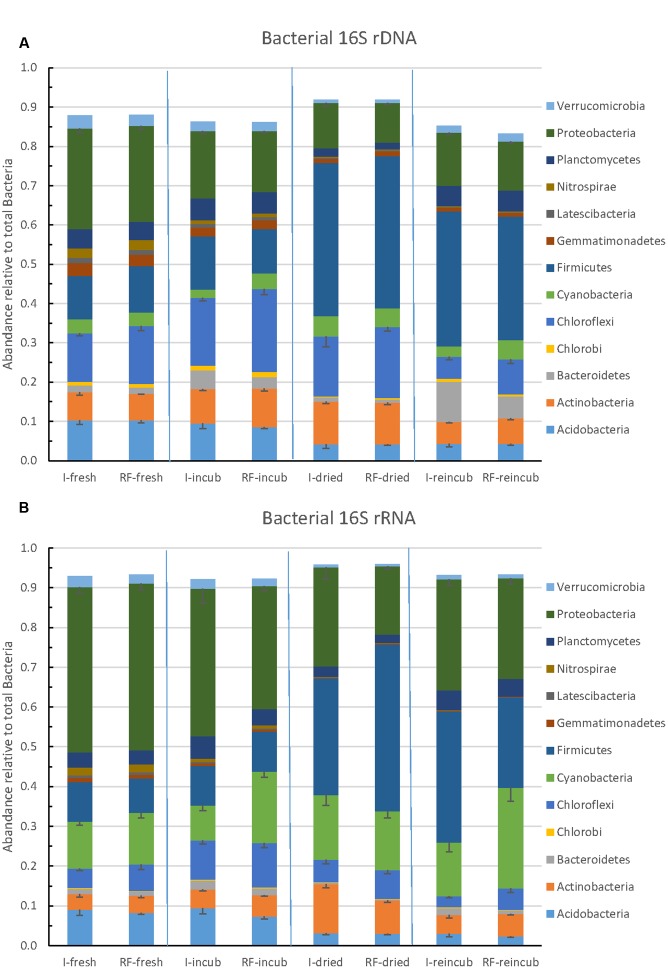
**Relative abundance of the predominant bacterial phyla in paddy soil from irrigated and rain-fed rice fields determined in fresh soil, after anaerobic incubation, after desiccation, and in the end of the subsequent anaerobic reincubation.** **(A)** 16S rRNA genes and **(B)** 16S rRNA. Mean ± SE, *n* = 4 different fields.

The community composition of Bacteria was determined by Illumina sequencing of bacterial 16S rRNA genes (DNA) and 16S rRNA (RNA). The relative abundance of the major bacterial taxa representing together >85% of the total number of bacterial sequences obtained by Illumina sequencing are shown in **Figure 6A**. The most abundant bacterial phyla were the *Firmicutes, Proteobacteria, Chloroflexi, Actinobacteria, Acidobacteria* and *Bacteroidetes*. There was little difference between samples from irrigated versus rain-fed fields, but community composition changed upon incubation, desiccation and rewetting. Whereas *Proteobacteria* was the most abundant phylum in the beginning, *Firmicutes* was the most abundant one in the end. A similar pattern was obtained on the level of ribosomal RNA (**Figure 6B**). On the RNA level, however, *Cyanobacteria* exhibited a much higher relative abundance (13–21% of total bacterial ribosomal RNA) than on the DNA level (2–4% of total bacterial ribosomal genes). The *Cyanobacteria* were mainly composed of Subsection IV and unclassified cyanobacteria (Supplementary Figure S9).

**FIGURE 7:**
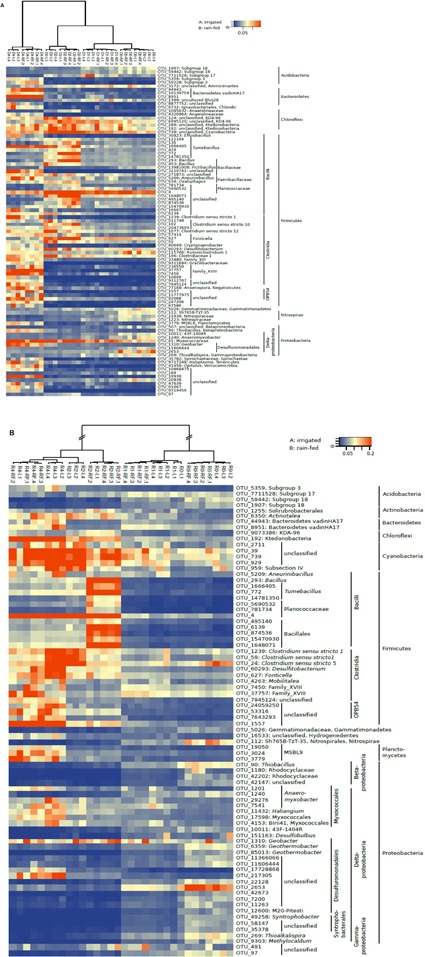
**Heatmap of the most relevant bacterial OTUs derived from** **(A)** 16S rDNA (labeled D) and **(B)** 16S rRNA (labeled R) extracted from soil of 4 replicate irrigated (labeled I.1–I.4) and rain-fed (labeled RF.1–RF.4) rice fields; in fresh soil (D0 or R0), after anaerobic incubation (D1 or R1), after desiccation (D2 or R2), and after anaerobic reincubation (D4 or R4). The colored scale gives the percentage abundance of OTUs relative to total Bacteria.

The similarity between the bacterial communities of irrigated and rain-fed soils was confirmed by NMDS analysis of bacterial OTUs (**Figure 4B**). However, OTUs derived from 16S rRNA genes were separated from those derived from ribosomal RNA. NMDS also showed a trend of the community compositions from fresh soil via incubated, desiccated to reincubated soil (**Figure 4B**). This trend was also seen in heatmaps of the bacterial OTUs, both on DNA (**Figure 7A**) and RNA level (**Figure 7B**). Similarly as for Archaea, the abundance of individual bacterial populations (OTUs on DNA level) changed most drastically after reincubation of desiccated soil (**Figure 7A**), while the abundance of bacterial ribosomal RNA changed already after the desiccation (**Figure 7B**). There, the most dramatic increases in relative abundance were seen among the *Firmicutes*, *Cyanobacteria*, *Planctomycetes* and several groups of *Deltaproteobacteria*, while several other OTUs among the *Proteobacteria* strongly decreased in relative abundance (**Figure 7B**). A similar picture was obtained on the level of DNA, but *Bacteroidetes* also increased during reincubation (**Figure 7A**).

The *Firmicutes*, which increased in relative abundance from the fresh to the desiccated and rewetted soils, consisted mainly of the class *Clostridia* (mainly the families of Clostridiaceae-1 and Family-XVIII), and only in the dry soil after desiccation the class *Bacilli* (mainly *Bacillales* and *Planococcaceae*) was dominant (**Figure 7B**). Cyanobacterial OTUs only became prevalent after desiccation and only on RNA level (**Figure 7B**). They belonged to unclassified taxa (**Figure 7B** and Supplementary Figure S9).

## Discussion

### Difference between Irrigated and Rain-Fed Soil and the Effect of Desiccation Stress

The results of NMDS analyses of Illumina sequences (**Figure 4**) and T-RFLP patterns (Supplementary Figures S5, S8) showed that the microbial communities in the paddy soils from irrigated versus rain-fed rice fields were not significantly different. The same was the case for abundances of Archaea and methanogens (*mcrA*), as well as CH_4_ production rates (**Figures 1**, **2**). Abundances of Bacteria were only slightly (but significantly) different. There were only slight differences in stable isotope fractionation caused by the relative contribution of hydrogenotrophic versus aceticlastic methanogenesis. Since both types of rice fields were established on the same soil series, we tentatively conclude that structure and function of the methanogenic microbial community was not affected by water management of the soil. This conclusion is tentative, since the detection of a difference may have been occluded by the flood catastrophe, which affected both field types in the year before sampling.

In the soils from both types of rice fields, desiccation stress followed by reflooding caused dramatic changes in structure and function of the methanogenic microbial communities. These conditions resulted in stimulation of CH_4_ production rates (**Figure 1**) and of transcription of *mcrA* (**Figure 2**) indicating expression of methanogenic activity, while the relative contribution of hydrogenotrophic methanogenesis was similar in both soil types, accounting for about 50% of total CH_4_ production. Desiccation and reflooding also affected the composition of the archaeal and bacterial communities both on the level of DNA (rRNA and *mcrA* genes) and of RNA (rRNA and *mcrA* transcripts) (**Figure 4** and Supplementary Figures S5, S8). Reaction upon desiccation stress was also observed in previous studies of rice fields, upland soils and lake sediments (Conrad et al., 2014b; Ji et al., 2015; Hernández et al., 2017). However, there were differences with respect to the microbial taxa affected and the ensuing function.

### Response of the Methanogenic Archaeal Community

Among Archaea, desiccation and reflooding resulted in decrease of the relative abundance of *Methanomicrobiales* and increase of *Methanocellales* both on DNA and RNA level (**Figure 3**). However, these two methanogenic taxa were generally less than 10% of the total archaeal abundance. More important were the *Methanobacteriales, Methanosarcinaceae* and *Methanosaetaceae*. On DNA level, these taxa exhibited a fairly constant relative abundance except in freshly dried soil where *Methanobacteriales* dominated. On RNA level, however, *Methanosarcinaceae* clearly increased in relative abundance upon desiccation and rewetting (**Figure 3**). There were also changes in the composition of the individual methanogenic orders and families, as seen from analysis of OTUs (**Figure 5**). Here OTUs within *Methanosarcinaceae*, *Methanobacteriales*, *Methanocellales* and *Methanomassiliicoccus* increased, while those within *Methanosaetaceae* either increased or decreased. In general, however, it was mainly the group of potentially aceticlastic methanogens (*Methanosarcinaceae, Methanosaetaceae*) that increased upon desiccation, also confirmed by T-RFLP analysis of *mcrA* genes and transcripts (Supplementary Figures S6, S7).

Stimulation of transcription in *Methanosarcinaceae* and/or *Methanosaetaceae* has also been observed when rice field soil had been treated with oxygen (Yuan et al., 2011) or when Chinese rice fields had been drained (Ma et al., 2012). The change in transcripts was generally more pronounced than that of the genes, indicating that activation of existing methanogens was faster and stronger than turnover of the microbial populations. However, aerated upland soils preferentially support the growth of *Methanosarcinaceae* and *Methanocellales* upon flooding (Angel et al., 2011; Ji et al., 2015; Hernández et al., 2017). These two groups were also found to become relatively abundant when rice fields underwent frequent drainage or crop rotation between rice and upland crops (Watanabe et al., 2006; Fernandez Scavino et al., 2013; Breidenbach and Conrad, 2015), or when lake sediments were dried and rewetted (Conrad et al., 2014b). All these observations support the concept that these two groups of methanogens are especially well adapted to desiccation and aeration stress (Conrad et al., 2006; Angel et al., 2012a; Lyu and Lu, 2015) and therefore, prevail in environments that undergo desiccation or aeration stress.

Changes in the community composition on the population level are often minor in rice field soils. The microorganisms persist even beyond drainage and seasonal fallow periods and maintain a rather diverse methanogenic community usually consisting of *Methanocellales, Methanosarcinaceae, Methanosaetaceae, Methanobacteriales* and *Methanomicrobiales* (Watanabe et al., 2006; Fernandez Scavino et al., 2013; Breidenbach and Conrad, 2015). Nevertheless, we found slight changes that only became evident when the community composition was analyzed on the taxonomic levels of genera and “species” (i.e., OTUs, 97% cut-off). Here, we saw for example that desiccation favored the genus *Methanomassiliicoccus*, a member of the recently discovered order *Methanomassiliicoccales* (Paul et al., 2012; Borrel et al., 2013). The few isolates of this order produce CH_4_ by reducing methanol with H_2_. It is unclear why they became favored by desiccation and rewetting.

In addition to non-methanogenic Thaumarchaeota, the Thai paddy soils also contained Woesearchaeota and MEG. These two phyla probably do not contain methanogenic microbes. However, future genomic analyses may provide surprises, as putative methanogenic activity was detected in phyla that were previously believed to be non-methanogenic (Evans et al., 2015; Nobu et al., 2016). MEG, previously classified as Rice Cluster 5 (Grosskopf et al., 1998) or LDS (Glissmann et al., 2004) were recently classified as Microarchaeota-Diapherotrites within the DPANN superphylum (Castelle et al., 2015; Ortiz-Alvarez and Casamayor, 2016). The environmental preferences of Woesearchaeota and MEG are unclear, but they were more frequently found in aquatic than in soil environments (Ortiz-Alvarez and Casamayor, 2016). In the Thai paddy soils the relative abundances of Woesearchaeota and MEG did not react upon desiccation and reflooding, implying that they were not actively reacting on the stress.

### Response of the Bacterial Community

The bacterial community is important for hydrolysis and fermentation of organic matter to eventually produce methanogenic substrates such as H_2_ and acetate (Conrad, 2007). Desiccation and flooding resulted in both soil types in a strong increase in the relative abundance of *Firmicutes*, especially of *Clostridia*, which was seen on RNA as well as DNA levels (**Figure 6**). The *Clostridia* replaced *Proteobacteria*, which decreased in relative abundance. *Chloroflexi* also decreased, while *Cyanobacteria* and *Bacteroidetes* increased, but the former only on RNA level and the latter only on DNA level. This pattern was also seen among the OTUs, where members of the phyla mentioned above were stimulated usually both on DNA and RNA levels. Active *Bacilli* were mainly prevalent immediately after desiccation, but decreased again after reflooding (**Figure 7B**). Among the *Proteobacteria* it was mainly members of *Deltaproteobacteria* that reacted to the stress conditions, with the relative abundance of some OTUs increasing and others decreasing (**Figure 7**).

A pronounced stimulation of *Firmicutes*, *Clostridia* in particular, after desiccation stress has also been observed in methanogenic Amazonian lake sediments (Conrad et al., 2014b), but was not observed in rice field and upland soils from the Sanjiang area in China (Hernández et al., 2017). Relative abundance of *Firmicutes* was also not enhanced in rice fields undergoing rotation with upland crops (Breidenbach et al., 2016a). In general, bacterial communities in rice fields seem to be relatively stable against water management (Itoh et al., 2013; Lopes et al., 2014; Zhao et al., 2014), although incubated rice soils show a change of the bacterial community structure with time of flooding and differences between oxic and anoxic zones of the soil (Noll et al., 2005; Shrestha et al., 2007; Rui et al., 2009; Kim and Liesack, 2015). Oxic and anoxic zones of rice field soil were also functionally different as seen by metatranscriptome analysis (Shrestha et al., 2009; Kim and Liesack, 2015). Oxic and anoxic incubation of rehydrated microbial mats also resulted in different patterns of bacterial community succession as seen by rRNA synthesis from ^18^O-labeled water (Angel and Conrad, 2013). These studies showed a dominance of *Firmicutes* (*Clostridia*) under anoxic and *Cyanobacteria* under oxic conditions.

Our observation that *Cyanobacteria* became active after desiccation stress of the Thai paddy soil is interesting, since incubation conditions were anoxic except during the desiccation period and soil was never exposed to light. This was in contrast to the studies of Kim and Liesack (2015), who assayed oxic surface soil of rice microcosms incubated in the greenhouse and those of Angel and Conrad (2013), who incubated microbial mats under oxic conditions in the light. We have presently no conclusive explanation for the stimulation of cyanobacterial activity after desiccation stress of Thai paddy soil.

*Clostridia* populations and activities were strongly enhanced by desiccation stress of the Thai paddy soil. Such stimulation is reasonable, since *Clostridia* are Gram-positive endospore forming bacteria that are well adapted to survive desiccation and can rapidly reactivate upon reflooding. Since they are also important agents of hydrolysis and fermentation of organic matter under anoxic conditions, it is plausible that they constitute one of the dominant bacterial groups. The question here is, why such stimulation has so far been found in the presently studied Thai soil and in Amazonian lake sediments, but not in flooded soils in general. In fact, rice fields usually contain *Firmicutes* only as minor populations making up less than 10% of the total Bacteria (Ahn et al., 2012; Cui et al., 2012; Xuan et al., 2012; Lee et al., 2015; Breidenbach et al., 2016a,b). The same is true for lake sediments (Wunderlin et al., 2014; Zhang et al., 2015; Dai et al., 2016). However, *Clostridia* populations can easily be enriched (Wunderlin et al., 2014), and such enrichment was indeed observed after desiccation in the Thai paddy and in Amazonian lake sediments. Possibly, such enrichment also happens in other environments. The question is when does it happen and when does it not happen.

We speculate that the quality of organic matter may be important. In the Thai paddy soils, CH_4_ production rates increased after desiccation indicating that organic matter decomposition was facilitated. The same happened in Amazonian lake sediments, where CH_4_ production was also increased after desiccation concomitant with an increase of *Clostridia* populations (Conrad et al., 2014b). We speculate that the quality of organic matter facilitated degradation after the desiccation period. In most paddy soils, however, CH_4_ production decreased after desiccation indicating that in these soils the organic matter became less degradable after desiccation (Ji et al., 2015; Hernández et al., 2017). In paddy soils, *Clostridia* dominate the early succession of anoxic systems but become less abundant and less active at later stages, probably since activities required for degradation of polysaccharide change with the depletion of available organic matter (Noll et al., 2005; Wegner and Liesack, 2016). If organic matter is eventually less available in paddy soils than in lake sediments, this difference may explain the different patterns of CH_4_ production and bacterial community shifts after desiccation. It would also signify that the Thai paddy soils behaved more similarly to a lake sediment than to other paddy soils. This assumption is consistent with the occurrence of Woesearchaeota and MEG archaea in the Thai soil, albeit they are not expected in soil environments. Another feature is the fraction of hydrogenotrophic methanogenesis, which after desiccation was comparable to the relatively high values (*f*_H2_ > 0.5) typically found in lake sediments (Conrad et al., 2011; Ji et al., 2016), while those in paddy soils are usually lower (*f*_H2_ < 0.4) (Yao and Conrad, 2000). Finally, we don’t know to which extent the resemblance of Thai paddies to lake sediments was effected by the flood catastrophe the year before sampling.

## Conclusion

Our study showed that irrigated and rain-fed rice fields in Central Thailand exhibited no major differences in function and structure of the methanogenic microbial communities. Differences were hypothesized because of different histories of the extents of drainage and flooding in the two field types, which likely affect the anaerobic bacteria and archaea involved in CH_4_ production. Our study also showed that the methanogenic microbial communities in both field types were similarly affected by desiccation stress. Such stress resulted in increased rates of CH_4_ production and affected the composition of the archaeal and bacterial communities, especially those with active transcription of the 16S rRNA and *mcrA* genes, but caused no changes in the absolute abundance of these microorganisms, except the transcription of *mcrA* genes, which increased. Desiccation enhanced particular groups among the Bacteria as well as the methanogenic Archaea. Some of these groups have previously been shown to withstand desiccation well, e.g., *Clostridia* and *Methanosarcinaceae*.

## Author Contributions

AR and MH: conducted and evaluated the experiments, prepared figures and tables, contributed equally. MK: conducted some experiments. AC and MY: organized and supervised sampling tour. RC: designed the study, wrote the manuscript.

## Conflict of Interest Statement

The authors declare that the research was conducted in the absence of any commercial or financial relationships that could be construed as a potential conflict of interest.

## Footnotes

1 http://www.r-project.org
