# Supplementary material for: Response of Methanogenic Microbial Communities to Desiccation Stress in Flooded and Rain-Fed Paddy Soil from Thailand
Source: Front Microbiol. 2017 May 5;8:785. doi: 10.3389/fmicb.2017.00785 (PMC5418361; doi:10.3389/fmicb.2017.00785)

Response of methanogenic microbial communities to desiccation stress in flooded and rain-fed paddy soil from Thailand

Andreas Reim[†], Marcela Hernández[†], Melanie Klose, Amnat Chidthaisong, Monthira Yuttitham, Ralf Conrad

Supplemental Material

APPENDIX S1

Experimental Procedures

Terminal restriction fragment length polymorphism (T-RFLP)

The analysis of terminal restriction fragment length polymorphism (T-RFLP) was carried out as described previously by using the following primer combinations: for archaeal 16S rRNA genes Ar109f (5′-ACKGCTCAGTAACACGT-3′) and Ar915r (5′-GTGCTCCCCCGCCAATTCCT-3′) (Chin et al., 1999; Grosskopf et al., 1998), with the reverse primer labelled with FAM (6-carboxyfluorescein); and for methanogenic *mcrA* genes mcrF (5′-GGTGGTGTMGGATTCACACARTAYGCWACAGC-3′) and mcrR (mcrR 5′-TTCATTGCRTAGTTWGGRTAGTT-3′) (Springer et al., 1995; Lueders & Friedrich, 2003), with the forward primer labelled with FAM. The 16S rRNA gene amplicons were digested with *TaqI* (Fermentas), the *mcrA* gene amplicons were digested with *Sau96I* (Fermentas), and the products were size-separated in an ABI 373 DNA sequencer (Applera, Darmstadt, Germany).

Cloning and sequencing

Clone libraries for archaeal 16S rRNA gene and mcrA genes were generated using the same primer sets as for T-RFLP (without FAM). PCR conditions were carried out as described previously (Lueders & Friedrich, 2003; Ke et al., 2014). For all samples, amplicons were purified by agarose gel electrophoresis using the GenElute Gel Extraction Kit (Sigma Aldrich). Cleaned PCR products were cloned into pGEM-T easy vector and transformed into competent *Escherichia coli* JM109 cells according to the manufacturer's recommendations (Promega). Cloned inserts were sequenced (Max-Planck Genome Centre, Köln, Germany) using the primer T7 (archaeal 16S rRNA and *mcrA* genes) and M13 (archaeal 16S rRNA gene) targeting the flanking region of the insert.

Taxonomy analysis

For archaeal 16S rRNA gene, reads were aligned against the SILVA 16S rRNA gene database using the naïve Bayesian classifier by using the mothur software platform (Schloss et al., 2009).

Accession numbers
Analyzed sequences for archaeal 16S rRNA gene and *mcrA* gene have been deposited in the GenBank database under the accession numbers KY851203 - KY851293 for archaeal 16S rRNA gene and accession numbers KY851117 - KY851202 for *mcrA* gene.

References

Chin, K.J., Lukow, T., Conrad, R. (1999) Effect of temperature on structure and function of the methanogenic archaeal community in an anoxic rice field soil. *Appl. Environ. Microbiol.* 65: 2341-2349.

Grosskopf, R., Stubner, S. and Liesack, W. (1998) Novel euryarchaeotal lineages detected on rice roots and in the anoxic bulk soil of flooded rice microcosms. *Appl. Environ. Microbiol.* 64**,** 4983-4989.

Ke, X., Lu, Y., Conrad, R. (2014) Different behaviour of methanogenic archaea and Thaumarchaeota in rice field microcosms. *FEMS Microbiol. Ecol.* 87, 18-29.

Lueders, T. and Friedrich, M. (2000) Archaeal population dynamics during sequential reduction processes in rice field soil. *Appl. Environ. Microbiol.* 66**,** 2732-2742.

Schloss, P. D., Westcott, S. L., Ryabin, T., Hall, J. R., Hartmann, M., Hollister, E. B. et al. (2009) Introducing mothur: open-source, platform-independent, community-supported software for describing and comparing microbial communities. Appl. Environ. Microbiol. 75, 7537-7541.

Springer, E., Sachs, M.S., Woese, C.R., Boone, D.R. (1995) Partial gene sequences for the A subunit of methyl-coenzyme M reductase (*mcrI*) as a phylogenetic tool for the family Methanosarcinaceae. *Int. J. Syst.Bacteriol.* 45: 554-559.

Table S1. Barcode identification for each of the samples analyzed. Raw data were deposited under the study accession number PRJNA362529 (Irrigated soils) and PRJNA362531 (Rain-fed soils) in the NCBI Sequence Read Archive (SRA). Partial 16S rRNA primers: F515 (5'-GTGCCAGCMGCCGCGGTAA), R806 (5'-GGACTACVSGGGTATCTAAT)

Sample	Template	Soil	Treatment	Barcode
D0A.1	DNA	Irrigated	Original soil	ACGTAC
D0A.2	DNA	Irrigated	Original soil	ACTGCA
D0A.3	DNA	Irrigated	Original soil	AGAGTC
D0A.4	DNA	Irrigated	Original soil	AGCTGA
D0B.1	DNA	Rain-fed	Original soil	AGTCAG
D0B.2	DNA	Rain-fed	Original soil	ATATCG
D0B.3	DNA	Rain-fed	Original soil	ATCGAT
D0B.4	DNA	Rain-fed	Original soil	ATGCTA
D1A.1	DNA	Irrigated	Incubated soil	CACAGT
D1A.2	DNA	Irrigated	Incubated soil	CAGTCA
D1A.3	DNA	Irrigated	Incubated soil	CATGAC
D1A.4	DNA	Irrigated	Incubated soil	CGATAT
D1B.1	DNA	Rain-fed	Incubated soil	CGCGCG
D1B.2	DNA	Rain-fed	Incubated soil	CGTATA
D1B.3	DNA	Rain-fed	Incubated soil	GACTAG
D1B.4	DNA	Rain-fed	Incubated soil	GAGATC
D2A.1	DNA	Irrigated	Dried soil	GATCGA
D2A.2	DNA	Irrigated	Dried soil	GTACAC
D2A.3	DNA	Irrigated	Dried soil	GTCACA
D2A.4	DNA	Irrigated	Dried soil	GTGTGT
D2B.1	DNA	Rain-fed	Dried soil	TACGTA
D2B.2	DNA	Rain-fed	Dried soil	TAGCAT
D2B.3	DNA	Rain-fed	Dried soil	TATACG
D2B.4	DNA	Rain-fed	Dried soil	TCAGAG

Continuation Table S1…

**Sample**	**Template**	**Soil**	**Treatment**	**Barcode**
D4A.1	DNA	Irrigated	Rewetted and incubated soil	ACGTAC
D4A.2	DNA	Irrigated	Rewetted and incubated soil	ACTGCA
D4A.3	DNA	Irrigated	Rewetted and incubated soil	AGAGTC
D4A.4	DNA	Irrigated	Rewetted and incubated soil	AGCTGA
D4B.1	DNA	Rain-fed	Rewetted and incubated soil	AGTCAG
D4B.2	DNA	Rain-fed	Rewetted and incubated soil	ATATCG
D4B.3	DNA	Rain-fed	Rewetted and incubated soil	ATCGAT
D4B.4	DNA	Rain-fed	Rewetted and incubated soil	ATGCTA
R0A.1	RNA	Irrigated	Original soil	CACAGT
R0A.2	RNA	Irrigated	Original soil	CAGTCA
R0A.3	RNA	Irrigated	Original soil	CATGAC
R0A.4	RNA	Irrigated	Original soil	CGATAT
R0B.1	RNA	Rain-fed	Original soil	CGCGCG
R0B.2	RNA	Rain-fed	Original soil	CGTATA
R0B.3	RNA	Rain-fed	Original soil	GACTAG
R0B.4	RNA	Rain-fed	Original soil	GAGATC
R1A.1	RNA	Irrigated	Incubated soil	GATCGA
R1A.2	RNA	Irrigated	Incubated soil	GTACAC
R1A.3	RNA	Irrigated	Incubated soil	GTCACA
R1A.4	RNA	Irrigated	Incubated soil	GTGTGT
R1B.1	RNA	Rain-fed	Incubated soil	TACGTA
R1B.2	RNA	Rain-fed	Incubated soil	TAGCAT
R1B.3	RNA	Rain-fed	Incubated soil	TATACG
R1B.4	RNA	Rain-fed	Incubated soil	TCAGAG

Continuation Table S1…

**Sample**	**Template**	**Soil**	**Treatment**	**Barcode**
R2A.1	RNA	Irrigated	Dried soil	ACGTAC
R2A.2	RNA	Irrigated	Dried soil	ACTGCA
R2A.3	RNA	Irrigated	Dried soil	AGAGTC
R2A.4	RNA	Irrigated	Dried soil	AGCTGA
R2B.1	RNA	Rain-fed	Dried soil	AGTCAG
R2B.2	RNA	Rain-fed	Dried soil	ATATCG
R2B.3	RNA	Rain-fed	Dried soil	ATCGAT
R2B.4	RNA	Rain-fed	Dried soil	ATGCTA
R4A.1	RNA	Irrigated	Rewetted and incubated soil	CACAGT
R4A.2	RNA	Irrigated	Rewetted and incubated soil	CAGTCA
R4A.3	RNA	Irrigated	Rewetted and incubated soil	CATGAC
R4A.4	RNA	Irrigated	Rewetted and incubated soil	CGATAT
R4B.1	RNA	Rain-fed	Rewetted and incubated soil	CGCGCG
R4B.2	RNA	Rain-fed	Rewetted and incubated soil	CGTATA
R4B.3	RNA	Rain-fed	Rewetted and incubated soil	GACTAG
R4B.4	RNA	Rain-fed	Rewetted and incubated soil	GAGATC

Sample location

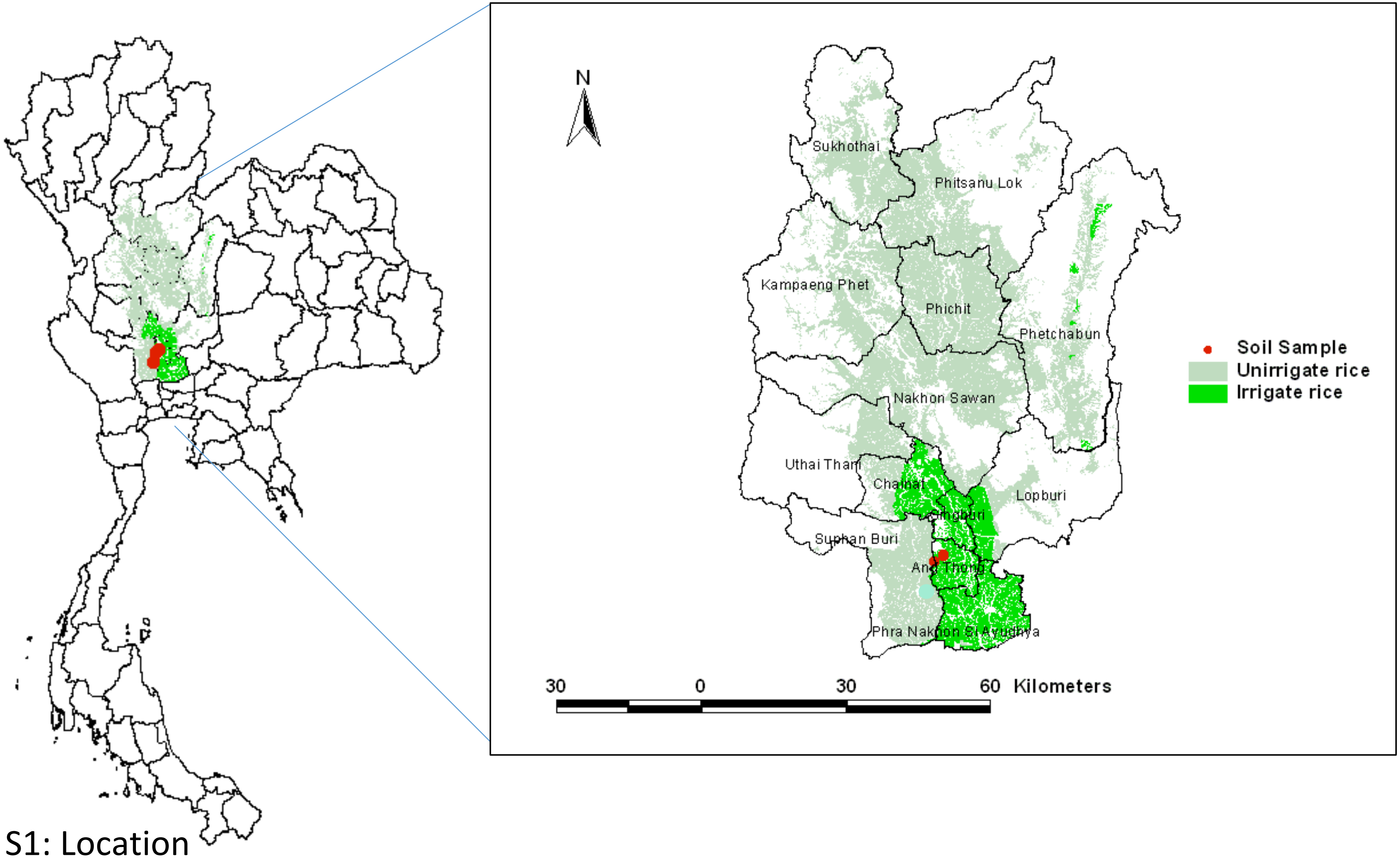

Fig. S1: Location

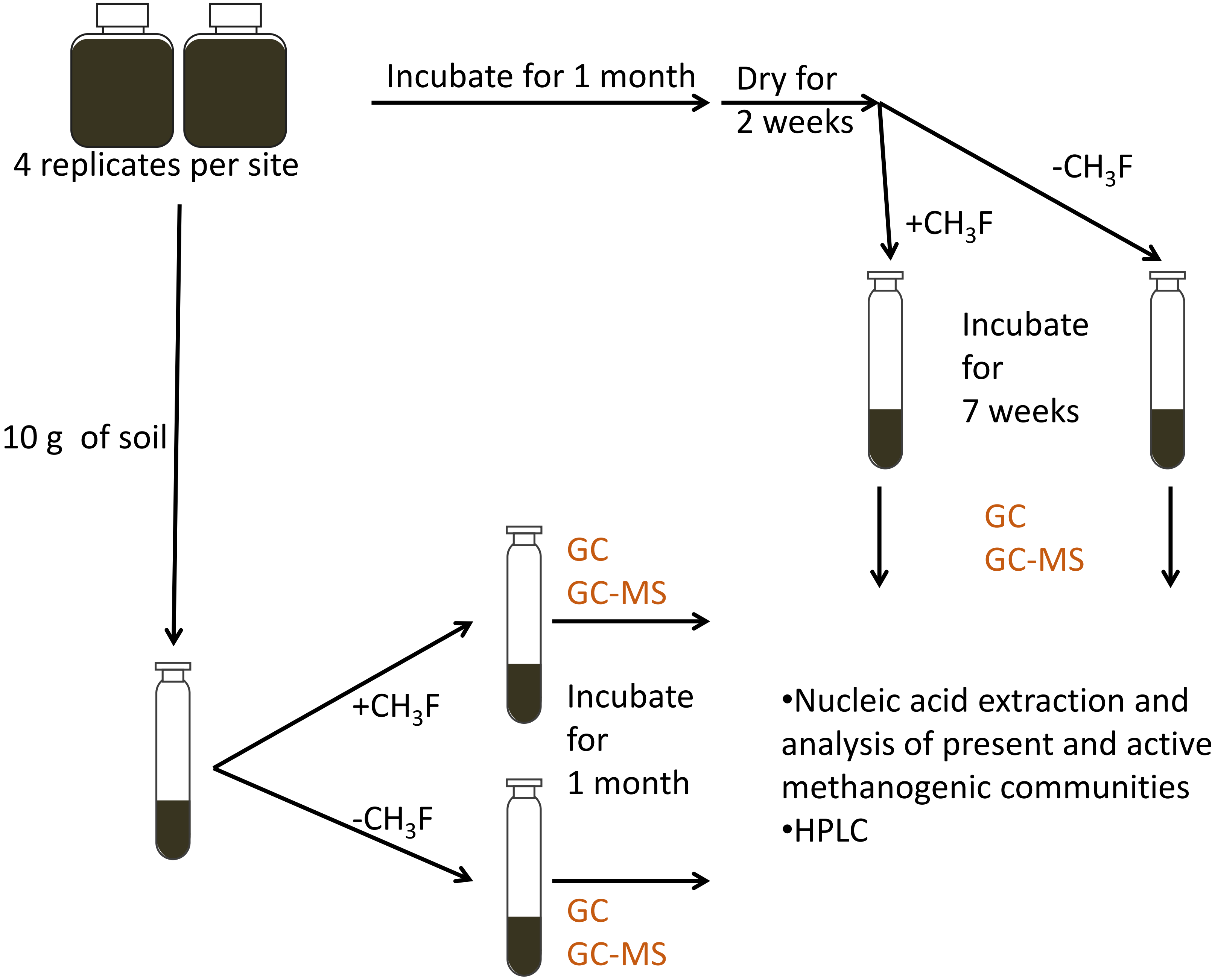

Fig. S2: Incubation scheme

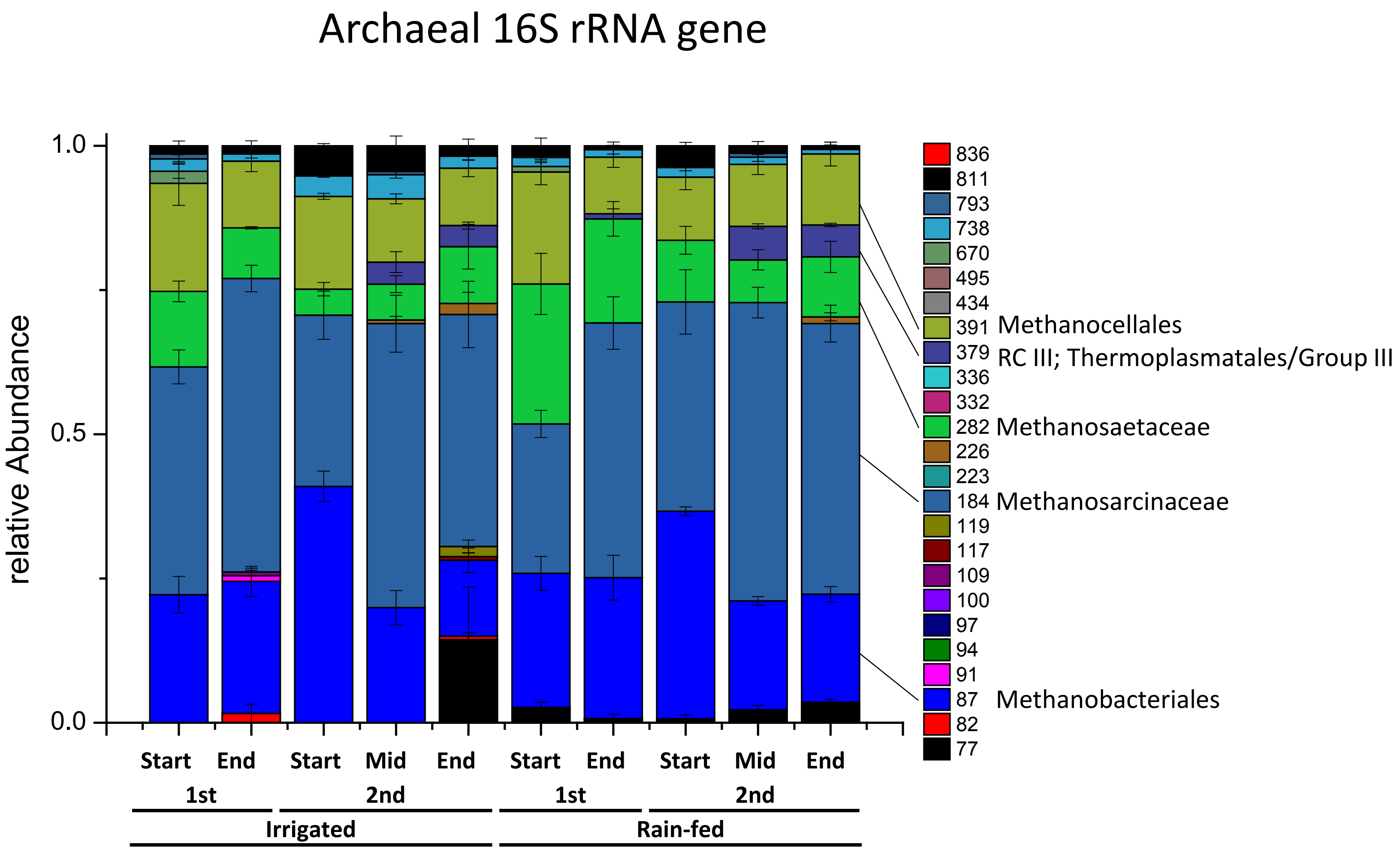

Fig. S3: The samples were taken in from fresh soil and at the end of the anaerobic incubation, after desiccation and during (mid) and at the end of the reincubation (as shown by arrows in Fig. 1). The assignment of T-RFs to methanogenic taxa is based on literature data (described in *Global Change Biol. 14:657-669, 2008*) and by sequencing of 96 clones each of archaeal 16S rRNA and *mcrA* genes.

.

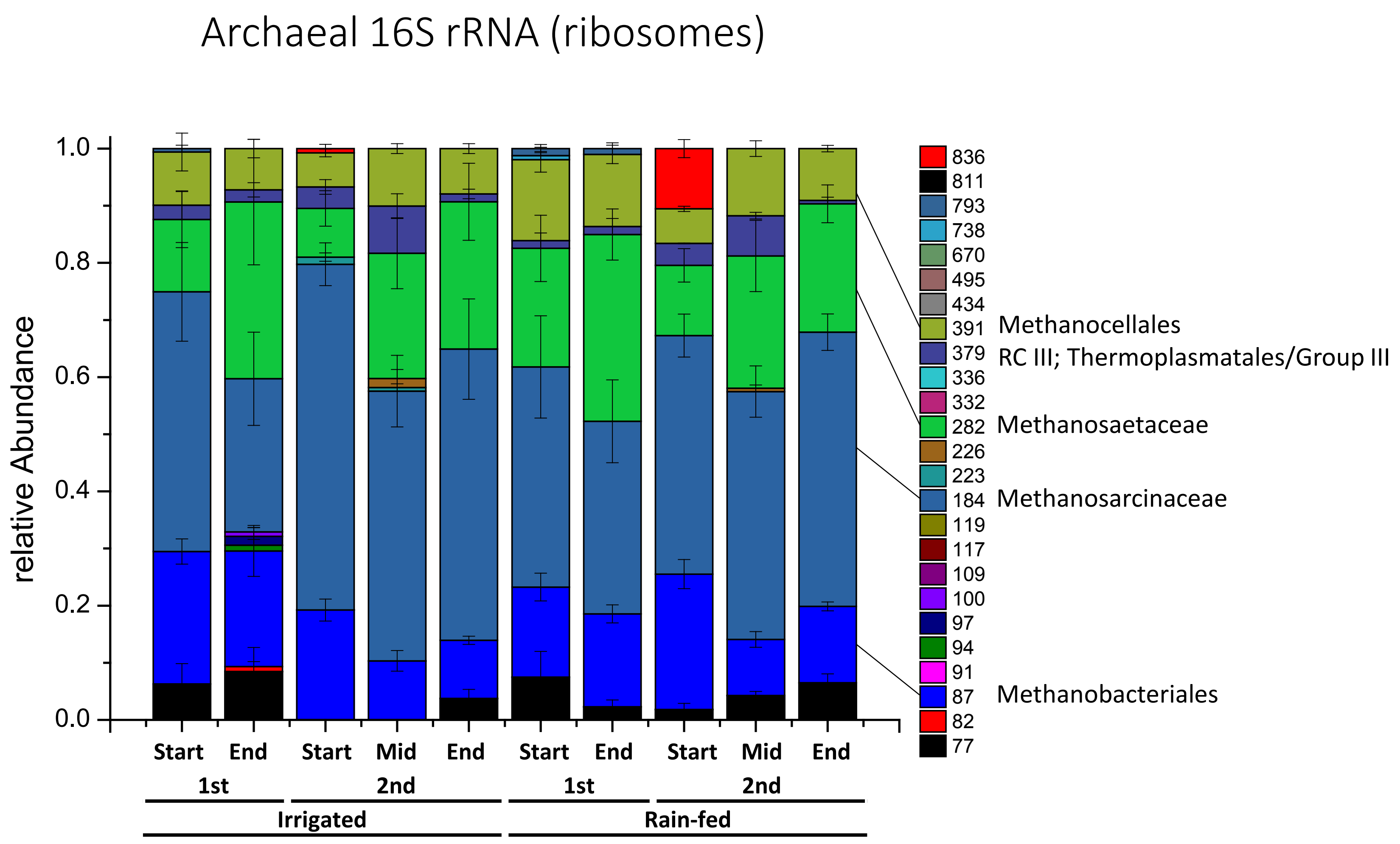

Fig. S4: The samples were taken in from fresh soil and at the end of the anaerobic incubation, after desiccation and during (mid) and at the end of the reincubation (as shown by arrows in Fig. 1). For assignment of T-RFs to methanogenic taxa see Fig. S3.

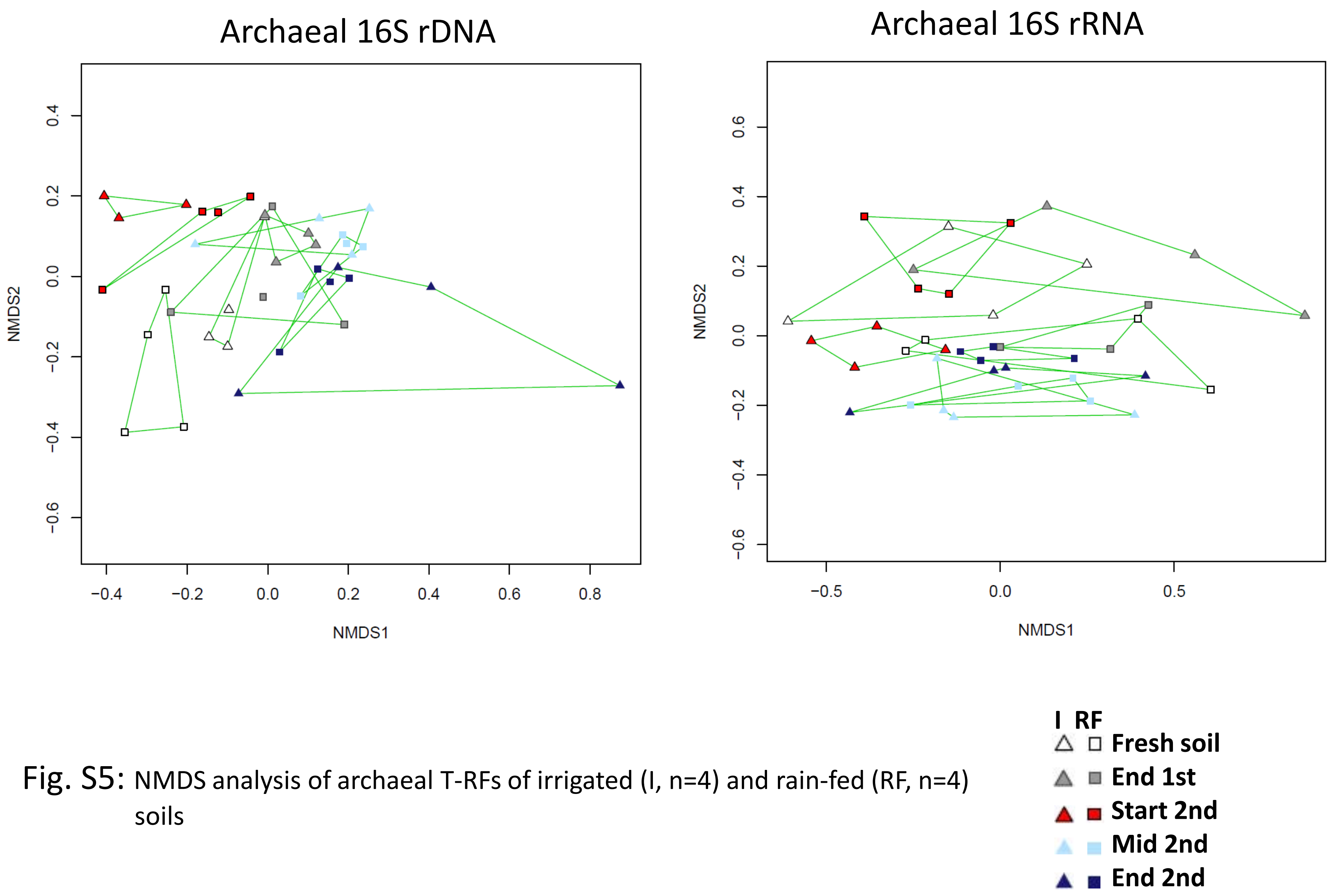

Fig. S5: NMDS analysis of archaeal T-RFs of irrigated (I, n=4) and rain-fed (RF, n=4) soils

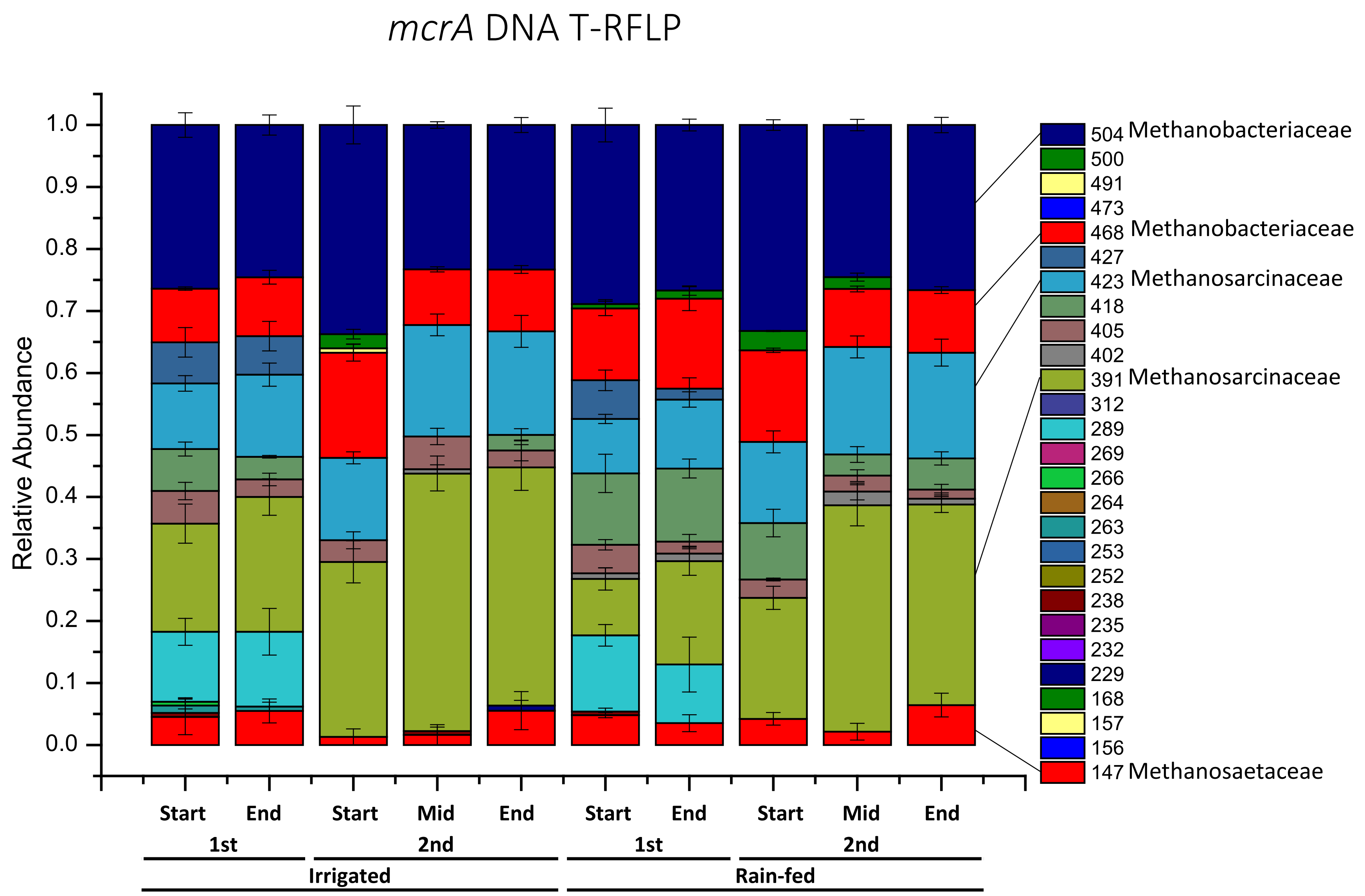

Fig. S6: The samples were taken in from fresh soil and at the end of the anaerobic incubation, after desiccation and during (mid) and at the end of the reincubation (as shown by arrows in Fig. 1). For assignment of T-RFs to methanogenic taxa see Fig. S3.

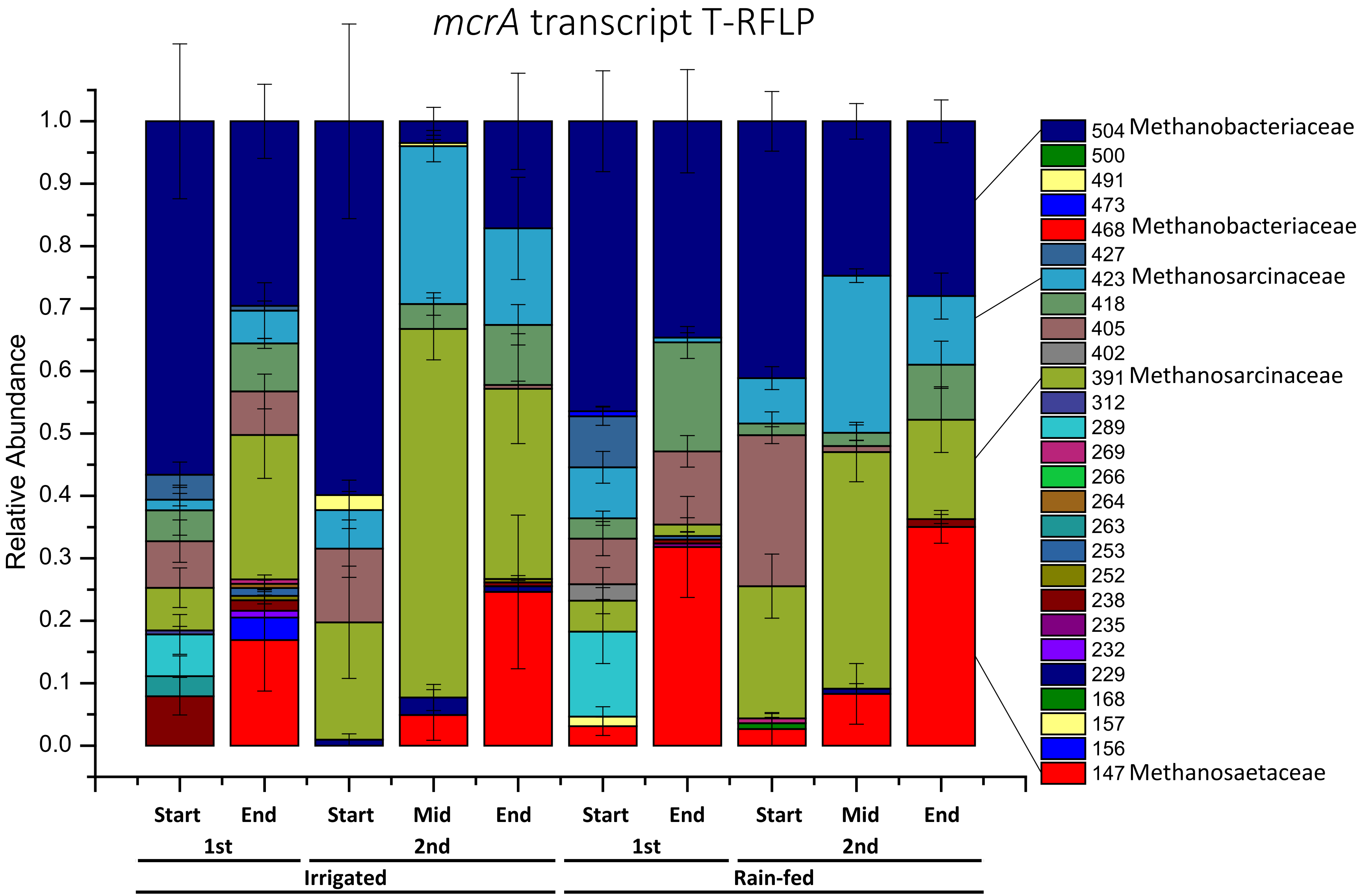

Fig. S7: The samples were taken in from fresh soil and at the end of the anaerobic incubation, after desiccation and during (mid) and at the end of the reincubation (as shown by arrows in Fig. 1). For assignment of T-RFs to methanogenic taxa see Fig. S3.

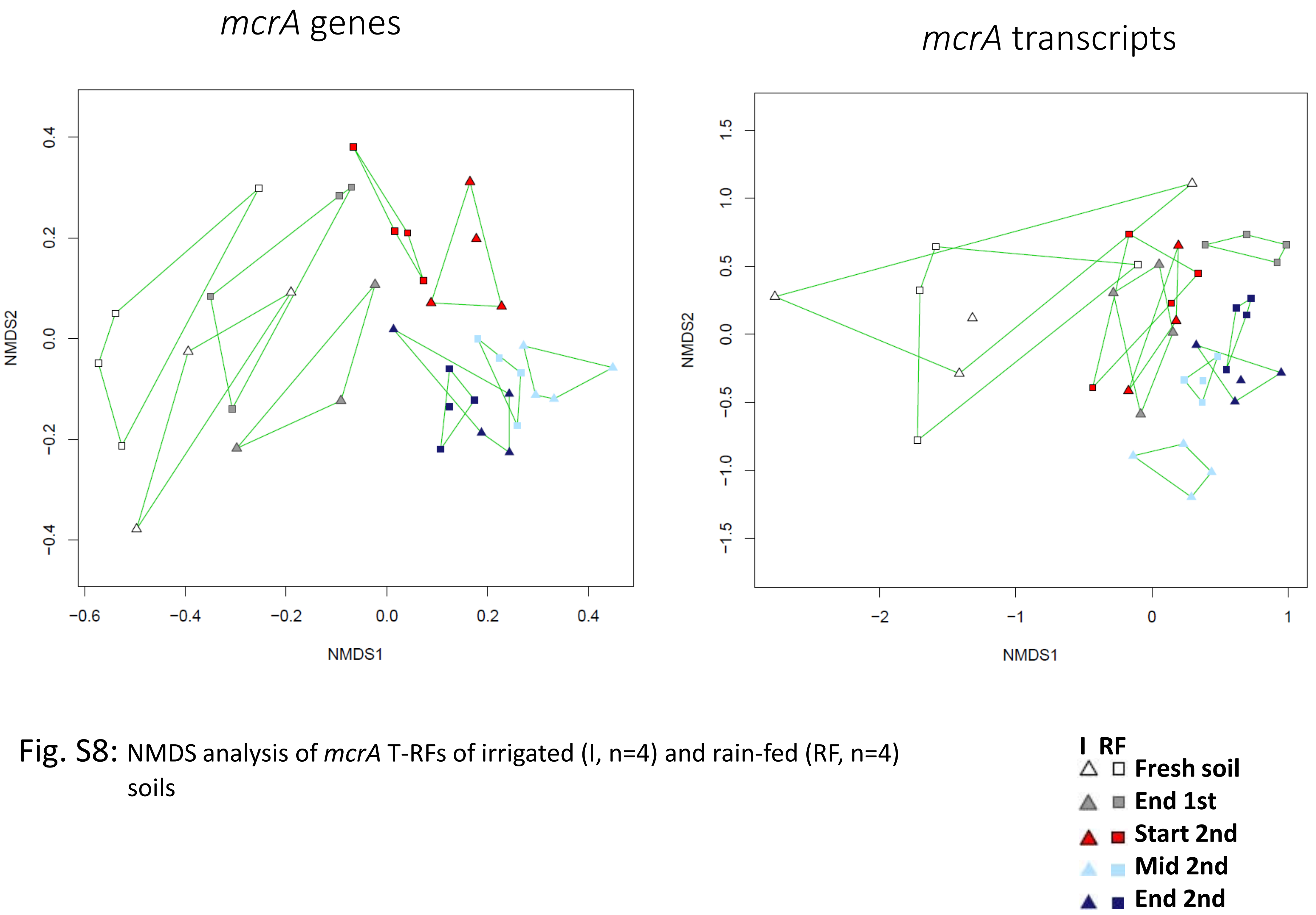

Fig. S8: NMDS analysis of *mcrA* T-RFs of irrigated (I, n=4) and rain-fed (RF, n=4) soils

Fig. S9:

Relative abundance of (A) genes and (B) RNA of bacterial 16S ribosomes affiliated to the phylum *Cyanobacteria,* as detemined from Illumina sequences

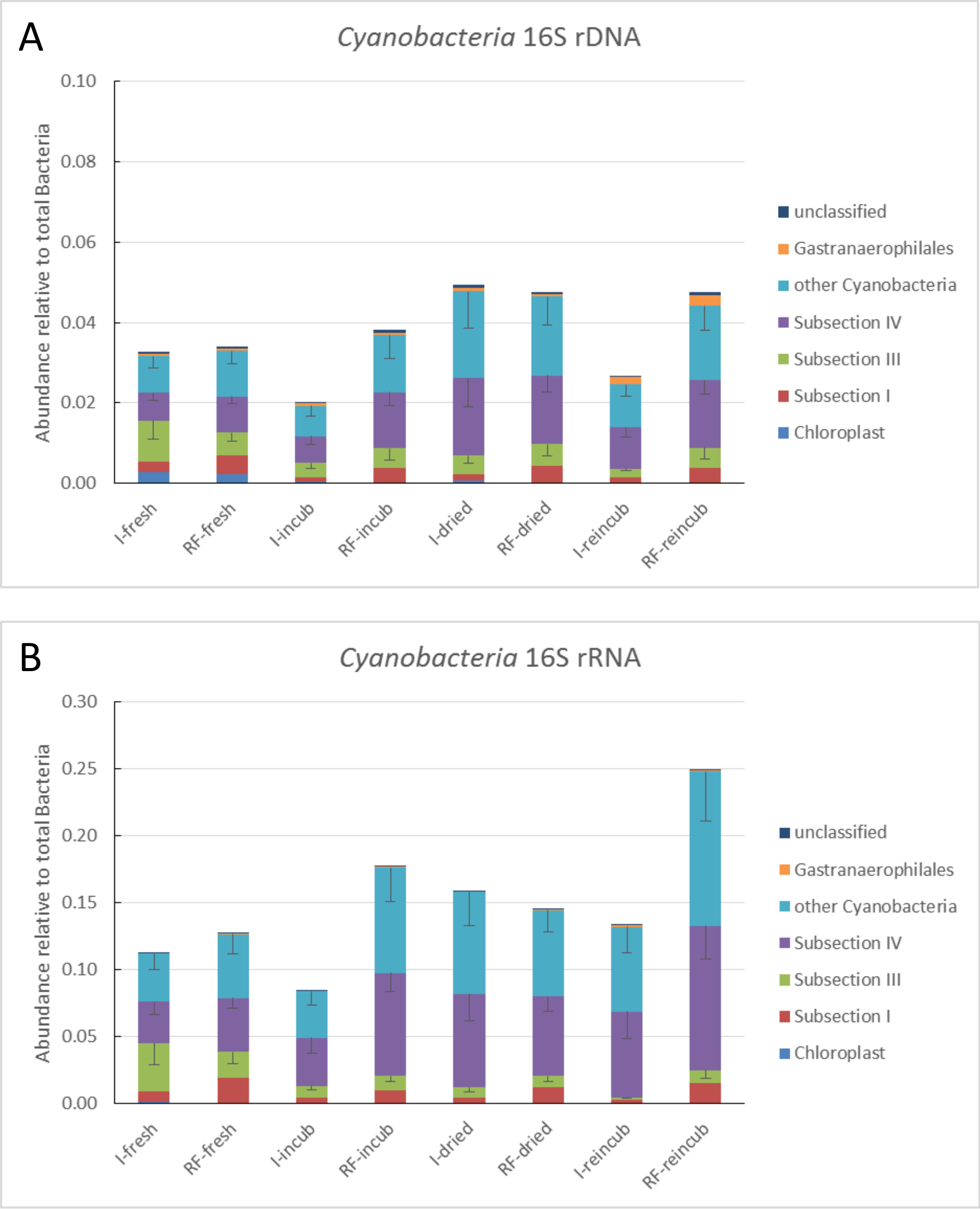

Supplement: Supplementary file 1 [file Presentation_1.pdf]
